# Rare Mixed Thyroid Carcinomas: A Report of Two Cases

**DOI:** 10.22038/IJORL.2021.48526.2614

**Published:** 2022-01

**Authors:** Nitya Subramanian, Sangeet Kumar Agarwal, Alok Agarwal, Pallav Gupta

**Affiliations:** 1 *Department of ENT, Sir Ganga Ram Hospital, New Delhi-110060.*; 2 *Department of Pathology, Sir Ganga Ram Hospital, New Delhi-110060.*

**Keywords:** Carcinomas, Immunohistochemistry, Thyroid

## Abstract

**Introduction:**

Thyroid cancers constitute 0.5%- 1% of all cancers of which differentiated cancers are most common. Mixed thyroid carcinomas are rare entities comprising only 0.5% of thyroid cancers. These tumours pose challenges in diagnosis by fine needle aspiration cytology.

**Case Report:**

Here, we present two rare cases of these mixed thyroid carcinomas. The first is that of a 21 year old female diagnosed with medullary thyroid carcinoma by the method of fine needle aspiration cytology (FNAC). She underwent definitive surgical treatment. Final histopathology revealed diagnosis of a nodular tumour with mixed features predominantly medullary carcinoma with areas of follicular carcinoma, confirmed on immunohistochemistry. Second report is that of a 45-year- old female diagnosed as medullary thyroid carcinoma on fine needle aspiration cytology (FNAC). Definitive surgical treatment was performed. Histopathology showed a mixed tumour with medullary and papillary components as confirmed through the process of immunohistochemistry.

**Conclusions:**

The knowledge of these rare carcinomas is important in avoiding a dilemma in management of this condition. Detection of these mixed tumours is difficult by FNAC. The definitive treatment essentially requires recognition of medullary component. We re-emphasize the importance of immune-histochemistry in arriving at an accurate diagnosis.

## Introduction

Thyroid malignancies constitute approximately 0.5% - 1% of all malignancies diagnosed every year. Differentiated carcinoma, including papillary and follicular carcinoma, is the most common malignancy affecting the thyroid gland whereas medullary thyroid cancer constitutes only 2-8% (1).

Mixed thyroid carcinomas are a rare entity comprising only 0.5% of all thyroid cancers (2). They are composed of neoplastic cells showing features of medullary carcinoma with positive immunoreactivity to calcitonin. These may be intermingled with either follicles, papillae, oxyphilic or solid areas with positive immunoreactivity to thyroglobulin. The first description of these mixed tumours was given by Hales et al in 1982, followed by Pfaltz in 1983(1).

Presence of two different malignant cells in one neoplasm poses a definite challenge in diagnosis by fine needle aspiration cytology, hence an uncertainty about the further treatment and prognosis. Here, we present two rare cases of mixed medullary thyroid carcinoma with concomitant follicular and papillary components, established through final histopathological examination.

## Case Report

Case 1

A 21-year-old female patient presented herself with a neck swelling of 6 months duration. She had no family history of thyroid cancers or MEN syndrome. Neck examination revealed a 2x2 cm midline thyroid swelling, which was mobile and moved on deglutition with no evidence of cervical lymphadenopathy. Fine needle aspiration of the thyroid swelling suggested a diagnosis of medullary carcinoma. Pre-operatively calcitonin was 150. Total thyroidectomy with comprehensive neck dissection was performed.

**Fig 1:**
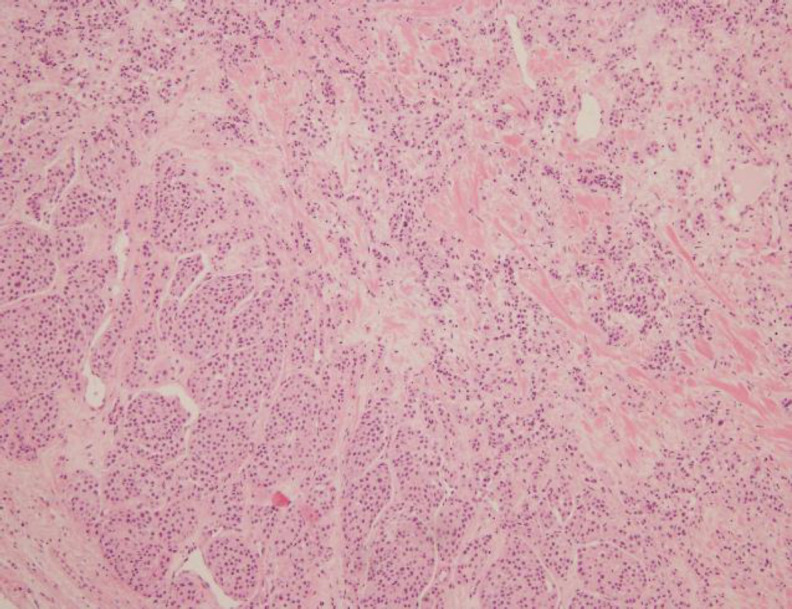
*Low power image showing co-existence of Papillary with medullary carcinoma*

Histopathological examination revealed a nodular tumour with monomorphic cells arranged in a nesting pattern with eosinophilic material resembling amyloid. On the periphery, few tumour nests showed micro-follicular cells with lympho-capsular invasion typical of follicular carcinoma, thus consistent with the diagnosis of mixed thyroid carcinoma comprising of medullary and follicular components. Presence of dual tumour population was confirmed through immunohistochemistry. The medullary component stained positive for calcitonin, synaptophysin and the follicular component showed positive staining for thyroglobulin. Refer fig. 1 showing HPE microscopic section indicating coexistence of follicular with medullary carcinoma.

Post-operative and at 1 year follow up calcitonin is in normal range and patient symptom free. 

Case 2

A 45-year-old female patient presented herself with a one-year history of slow growing swelling in the neck. She had no family history of thyroid cancers. Local examination revealed a solitary, well defined nodule in the left lobe of thyroid with no palpable cervical lymph nodes. Ultrasound of the neck showed evidence of a thyroid lesion with enlarged lymph nodes on the left side. Fine needle aspiration suggested a diagnosis of medullary thyroid carcinoma. Preoperatively CEA and Calcitonin were raised. 

**Fig 2:**
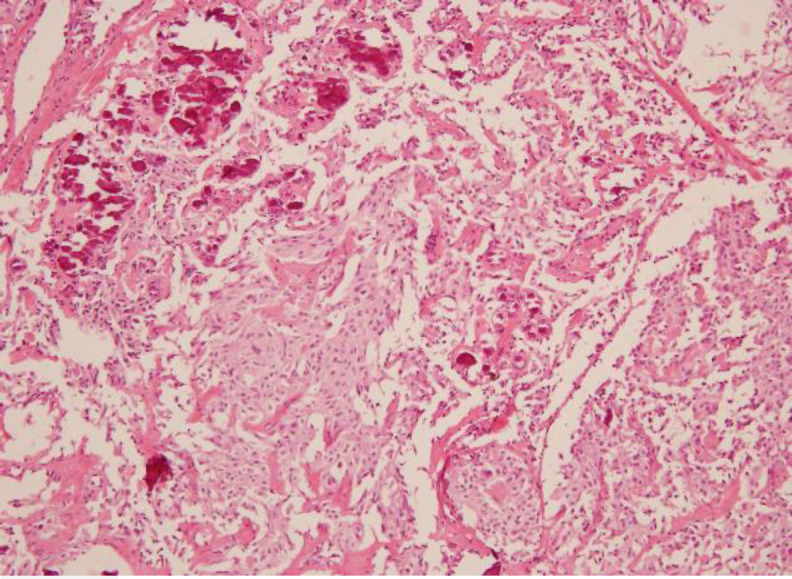
Low power image showing coexistence of follicular with medullary carcinoma

Total thyroidectomy with modified radical neck dissection with central compartment clearance was performed. On final histology, the appearance was typical of a medullary thyroid carcinoma with monomorphic cells in nesting and trabecular pattern. Focal amyloid deposition with positive congo red staining was seen. Immunostaining showed presence of tumour cells positive for calcitonin and chromogranin. A section from other side showed papillary structures lined by cells with overlapping nuclei with characteristic nuclear grooving, consistent with the diagnosis of papillary thyroid carcinoma. These tumour cells showed positive immune staining for thyroglobulin and CK-19 and negative for calcitonin. Refer fig. 2 showing microscopic picture of co- existence of Papillary with medullary carcinoma of Thyroid.

Post operatively calcitonin levels were normal and at 1 year follow up patient is asymptomatic.

## Discussion

Mixed thyroid cancers are very rare tumours that represent less than 0.5% of all thyroid malignancies. Fewer than 40 such cases have been reported so far (2). As defined by world health organisation, they are tumours that demonstrate morphological features of both medullary carcinoma (showing immunoreactivity to calcitonin) and follicular or papillary carcinomas (showing immunoreactivity to thyroglobulin) (3). 

These mixed tumours may be present either in a synchronous fashion where they appear as anatomically separate tumours or as two components mixed within a single lesion (4). Our cases showed both variants of these rare malignancies. In our first report, mixed tumour was identified with medullary and follicular carcinoma showing distinct immune histochemical differentiation. Presence of thyroglobulin positive neoplastic follicles formed a minor component of a predominant medullary carcinoma. In the second case presented, papillary and medullary carcinoma were present in a synchronous fashion each showing characteristic immunostaining.

The origin of these mixed thyroid carcinoma is not exactly established, yet one of the hypotheses shows that the tumour might arise from multipotent stem cells. An alternative hypothesis presumes a common oncogenic stimulus affecting both follicular and parafollicular cells (5).

Recognition of these mixed tumours is difficult by FNAC and leads to high probability of misdiagnosis (6). According to existing literature, the treatment of such tumours should be governed by its medullary component. Thus, the recognition of the medullary component of these mixed carcinomas by FNAC becomes very essential as the definitive surgical treatment for a medullary carcinoma includes not only thyroidectomy but a comprehensive neck dissection with no efficient adjuvant therapy (7). Even the prognosis of mixed thyroid carcinoma depends upon the medullary component. Thus, the prognosis is also worse as compared to a pure papillary or follicular carcinoma (2).

The knowledge of these rare carcinomas is important in avoiding a dilemma in further management. We re-emphasize the need to confirm the diagnosis by immune histochemistry in thyroid carcinomas to reach an accurate diagnosis.
